# Altered Small-World Brain Networks in Schizophrenia Patients during Working Memory Performance

**DOI:** 10.1371/journal.pone.0038195

**Published:** 2012-06-06

**Authors:** Hao He, Jing Sui, Qingbao Yu, Jessica A. Turner, Beng-Choon Ho, Scott R. Sponheim, Dara S. Manoach, Vincent P. Clark, Vince D. Calhoun

**Affiliations:** 1 The Mind Research Network, Albuquerque, New Mexico, United States of America; 2 Department of Electrical and Computer Engineering, University of New Mexico, Albuquerque, New Mexico, United States of America; 3 Department of Psychiatry, University of Iowa, Iowa City, Iowa, United States of America; 4 Minneapolis VA Health Care System and Department of Psychiatry, University of Minnesota, Minneapolis, Minnesota, United States of America; 5 Department of Psychiatry, Massachusetts General Hospital, Harvard Medical School, Charlestown, Massachusetts, United States of America; 6 Department of Psychology, University of New Mexico, Albuquerque, New Mexico, United States of America; 7 Departments of Psychiatry and Neurobiology, Yale University, New Haven, Connecticut, United States of America; 8 Olin Neuropsychiatry Research Center, Hartford, Connecticut, United States of America; Indiana University, United States of America

## Abstract

Impairment of working memory (WM) performance in schizophrenia patients (SZ) is well-established. Compared to healthy controls (HC), SZ patients show aberrant blood oxygen level dependent (BOLD) activations and disrupted functional connectivity during WM performance. In this study, we examined the small-world network metrics computed from functional magnetic resonance imaging (fMRI) data collected as 35 HC and 35 SZ performed a Sternberg Item Recognition Paradigm (SIRP) at three WM load levels. Functional connectivity networks were built by calculating the partial correlation on preprocessed time courses of BOLD signal between task-related brain regions of interest (ROIs) defined by group independent component analysis (ICA). The networks were then thresholded within the small-world regime, resulting in undirected binarized small-world networks at different working memory loads. Our results showed: 1) at the medium WM load level, the networks in SZ showed a lower clustering coefficient and less local efficiency compared with HC; 2) in SZ, most network measures altered significantly as the WM load level increased from low to medium and from medium to high, while the network metrics were relatively stable in HC at different WM loads; and 3) the altered structure at medium WM load in SZ was related to their performance during the task, with longer reaction time related to lower clustering coefficient and lower local efficiency. These findings suggest brain connectivity in patients with SZ was more diffuse and less strongly linked locally in functional network at intermediate level of WM when compared to HC. SZ show distinctly inefficient and variable network structures in response to WM load increase, comparing to stable highly clustered network topologies in HC.

## Introduction

Small-world networks strike a balance between high levels of local clustering and short path lengths linking all nodes even though most nodes are not neighbors of one another [1]. This optimized property offers a structural substrate for graph analysis on functional segregation and integration of the brain [2], [3], [4]. Network metrics such as efficiency provide a vital measure of how effectively information is passed and processed between different brain regions. Analysis of network organizational properties may also reveal disease-related abnormalities in functional brain networks among patients [5], [6], including schizophrenia (SZ) during resting state [7], [8], [9] as well as task-related data such as auditory oddball [10] and verbal memory [11]. In our study, we applied the small-world network analysis towards functional magnetic resonance imaging (fMRI) data collected during a working memory (WM) task.

WM is a construct that refers to maintaining and manipulating information on-line, in the mind’s eye in the service of guiding behavior. It is considered to be a temporary store whose contents are continually updated, scanned and manipulated in response to immediate processing demands [12]. WM deficits in SZ are consistently observed, relatively treatment-resistant and have been hypothesized to underlie many cognitive deficits and symptoms in SZ, manifested in longer reaction time and less accurate performance, especially as memory load increases [13], [14], [15]. They are accompanied by aberrant brain activation, particularly in the dorsolateral prefrontal cortex (DLPFC) [15], [16], [17]. The relation of working memory load or demand to DLPFC activation can be described as an inverted-U shaped function with activation increasing with increasing demand to the point that capacity is reached, at which point activation declines. In schizophrenia, this point is reached at a lower level of demand, and this hypothetical curve is shifted to the left, reflecting lower WM capacity [16], [18], [19]. In addition to DLPFC, WM performance is associated with activation in a network of brain regions [20], [21], [22], [23], as well as deactivation in the default mode network (DMN) [24]. Deficient WM in SZ is associated with aberrant activation in these networks [25], [26], [27], [28], [29].

Several studies utilizing graph analysis have investigated changes in functional network properties during WM tasks. For example, small-world structure has been reported in simultaneous MEG and EEG (MEEG) at different bands [30]. Analyses of EEG data demonstrate that optimal patterns are decreased or absent in SZ [31], [32]. Bassett and colleagues [33] reported that task performance correlated with global cost efficiency of the MEG beta-band network. An fMRI study in healthy subjects also showed that small-world network connectivity decreases as a function of increasing WM load [34]. These studies have utilized the n-back paradigm [35], which requires the temporal tagging and updating of information on each trial, and therefore has a very steep difficulty slope with increasing demand (i.e., 1 to 3 back) making it difficult to vary the load and stay within the capacity of SZ. As the Sternberg Item Recognition Paradigm (SIRP) [36] emphasizes the maintenance over the manipulation of information, the difficulty slope is less steep, allowing the parametric manipulation of load while staying within the WM capacity of SZ [26]. In a previous report on fMRI data collected during the SIRP [37], group independent component analysis (ICA) showed significant abnormalities in SZ relative to HC in both negatively task-correlated brain regions (DMN), and positively task-correlated areas (DLPFC). To our knowledge, no studies have evaluated network alterations in task-related brain regions in SZ during performance of a WM task with varying load.

The goal of this study is to investigate the topological properties in small-world networks derived from a data-driven (ICA defined) examination of task-elicited brain activity for both SZ and HC during the SIRP at three levels of WM load. We hypothesized that the functional network of task-related brain regions would change according to WM load in all subjects, and that SZ will show less efficient small-world network structures when compared to HC.

## Materials and Methods

### Ethics Statement

The Institutional Review Board at each site, the University of New Mexico and the University of Minnesota, approved this study, and all participants provided written informed consent. All patients recruited in the study had the capacity to consent, and showed reasonable performance during the task.

### Participants

A total of 35 patients with chronic SZ and 35 demographically matched HC were recruited and scanned from two sites, the University of Minnesota, and the University of New Mexico, as part of the Mind Clinical Imaging Consortium (MCIC) study. Those two sites were picked out of all four sites of MCIC study as subjects’ BOLD activation from these two had minimal site differences, and all subjects’ behavioral data were recorded. HC were free from any Axis I disorders as assessed with the Structured Clinical Interview for DSM-IV-TR (SCID) [38]. Patients met the criteria for SZ defined by the DSM-IV based on the SCID and review of the associated case files by experienced raters located with each site. All patients were stabilized on medication prior to the fMRI scan run. Handedness of subjects were determined by Edinburgh Handedness Inventory [39] and the education of subjects were evaluated by Wide Range Achievement Test (3rd ed.), Reading subtest (WRAT-3RT). Demographics and clinical characteristics of subjects are presented in Table 1.

**Table 1:** Demographics of Subjects and Symptom Scores for SZ.

	HC (n = 35)	SZ (n = 35)	p-value
**Age**	34.6±11.6, range 18–60	34.3±11.8, range 20–60	0.91
**Sex (Male/Female)**	27/8	26/9	0.82
**Handedness (non-right hand)**	3 left handed	1 left handed 3 ambidextrous	<0.0001
**Parental socioeconomic status**	2.4±0.6	2.6±1.0	0.19
**Education**	50.8±5.0	47.7±5.2	0.01
**Years since diagnosis**	n/a	13.1±11.1	
**Symtoms**	n/a	Positive = 5.0±2.2 negative = 7.5±3.1 disorganization = 1.9±2.2	

### Sternberg Item Recognition Paradigm

**Figure 1:**
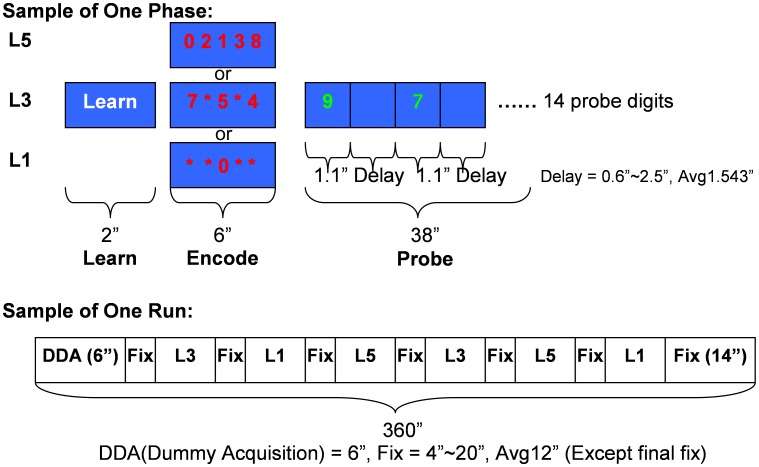
The SIRP timing and design. Top: Timing and contents of each Prompt-Encode-Probe block for each WM load level. Bottom: A sample run combining six blocks at different WM load levels in pseudo-random order.

The SIRP was adapted for fMRI as a block design divided into three runs. Each run contained two blocks of each of three WM load levels (Low Load: 1 digit (L1), Medium Load: 3 digits (L3), and High Load: 5 digits (L5)) presented in a pseudorandom order. As shown in Figure 1, each block began with a prompt that lasted for 2 seconds and displayed the word “Learn”. The learning prompt was followed by the encode condition of 6 seconds, which displayed the memory set of either one, three or five digits in red font (constituting the three levels of WM load) which the subjects needed to hold on-line in WM. This was followed by a series of 14 probes each consisting of a single digit in green font, each digit lasting 1.1 seconds. Half of the probes were targets (members of the memory set) and the other half were foils. The time between each probe digit pseudo-randomly varied from 0.6 to 2.5 seconds. The probe condition lasted for 38 seconds in total. For each probe digit subjects were required to indicate whether or not the probe was a target or foil by pressing a button with their right or left thumb (randomly assigned). There were fixation epochs between WM blocks that served as a baseline. Each run lasted 6 minutes.

Each subject was instructed to respond as quickly and accurately as possible. To mitigate motivational deficits subjects were given a bonus of 5 cents for each correct response, which was mailed to the participant after completion of the task.

The SIRP dataset this study utilized was also used as part of other MCIC studies [37], [40], [41], [42], [43], [44], [45] which used different analyses from what we presented here.

### Imaging Parameters

Among the two sites where data were collected, the University of New Mexico was using a Siemens Sonata 1.5T scanner, and the University of Minnesota was using a Siemens Trio 3.0T scanner. The echo planar imaging sequences were utilized and the pulse sequence parameters were almost the same: orientation  =  AC-PC line, number of slices  = 27, slice thickness  = 4 mm, slice gap = 1 mm, TR = 2000 ms, TE = 40 ms (1.5T scanner) or 30 ms (3T scanner), FOV = 22 cm, flip angle = 90°, matrix = 64×64, voxel dimension  = 3.4×3.4×4 mm^3^.

### Preprocessing

Data were preprocessed using the software package SPM5 (http://www.fil.ion.ucl.ac.uk/spm). Images were first realigned using a motion correction algorithm unbiased by local signal changes called INRIalign [46], [47]. The output of the realignment parameters from the SPM were kept as previous studies found functional connectivity of fMRI was sensitive to the head motion [48], [49].

A slice-timing correction was performed on the fMRI data after realignment to account for possible errors related to the temporal variability in the acquisition of the fMRI datasets. Data were spatially normalized [50] into the standard Montreal Neurological Institute space using an SPM5 echo-planar imaging (EPI) template and then spatially smoothed with a 9×9×9 mm^3^ full width at half-maximum Gaussian kernel. The data (originally collected at 3.4×3.4×4 mm^3^) was slightly subsampled to 3×3×3 mm^3^ (during normalization) resulting in 53×63×46 voxels. The time courses were then filtered with a Butterworth band-pass filter (0.003–0.23 Hz), to reduce drift effects and noise [4], [7], [51]. The frequency range of the filter was based on a factor of 0.01 to 0.9 multiplied by the Nyquist frequency of TR during the scanning (2000 ms, corresponding to 0.5 Hz). This cutoff range kept most of useful information during the scan, and did not filter out the task frequency, where encoding and probe processes last 6 seconds (0.167 Hz) and 38 seconds (0.026 Hz) respectively.

### Selection of Regions of Interest

The GIFT toolbox (http://icatb.sourceforge.net) was used to perform group spatial independent component analysis with infomax algorithm [52]. Time courses of three runs for each subject were temporally concatenated during the group ICA. The component number was set to be 26 as estimated by a modified minimum description length (MDL) criterion [53].

**Figure 2:**
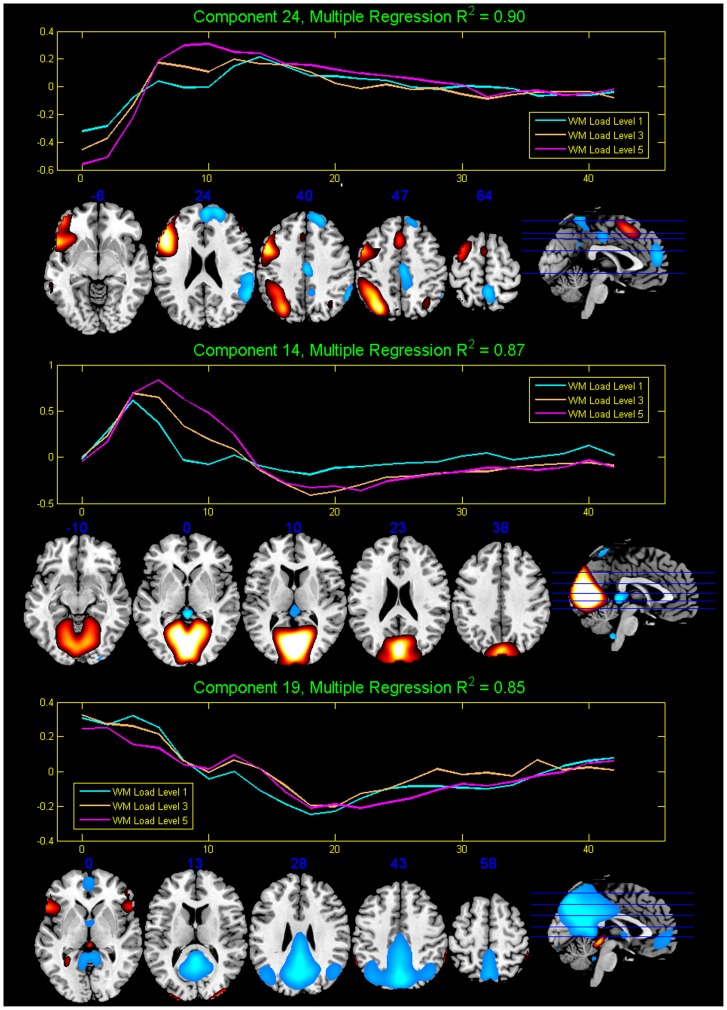
The 3 selected components and their averaged time courses from Group ICA.

Regressions were performed against the stimuli for each component, to get the weights (beta values) on each of the regressors. There were 12 regressors for each run, corresponding to two encodes and two probes for each of the three WM loads. To find the more task-related components, one sample t-tests were performed on the 12 beta values assessed from the regression. The components were sorted based on the p-value of t-test: the lower the p-value of the beta weights, the more task-related the component. For each of the 12 regressors, we listed the 5 components with the lowest p-values (10^−5^ – 10^−27^) in order to identify the most frequently occurring components among all regressors. Three common components were found as most task-related, i.e., Component 19, 14 and 24, as shown in Figure 2.

Specifically, component 24 overlapped the left DLPFC, consistent with demonstrated neural substrates of verbal WM tasks; component 14 was located in the bilateral occipital lobe, which is involved in visual perception; and component 19 overlapped regions found in the default mode network [54] including the posterior cingulate, precuneus, and cuneus. The three components were selected as regions of interest on which the small world network was implemented. Components 24 and 14 were positively correlated with presentation of the task stimuli, while component 19 was negatively correlated.

**Figure 3:**
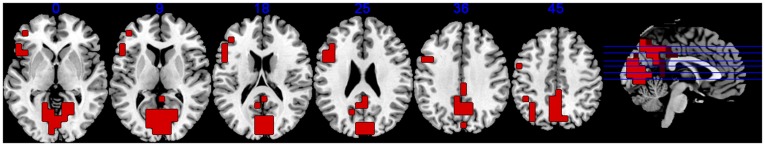
The spatial mask applied to build network.

The mask of regions of interest (ROIs) was generated by thresholding the spatial maps of the 3 selected components with |z|>2.0. ROI was then divided into 105 spatially adjacent 3×3×3-voxel sized blocks. Every block was then subsampled by averaging together, that is, the preprocessed BOLD signal of all voxels within a given spatial block were averaged into one time course, resulting 105 spatial blocks which were used to compute partial correlation below. The finial mask is shown in Figure 3.

### Dividing the Time Courses According to WM Load

**Figure 4:**
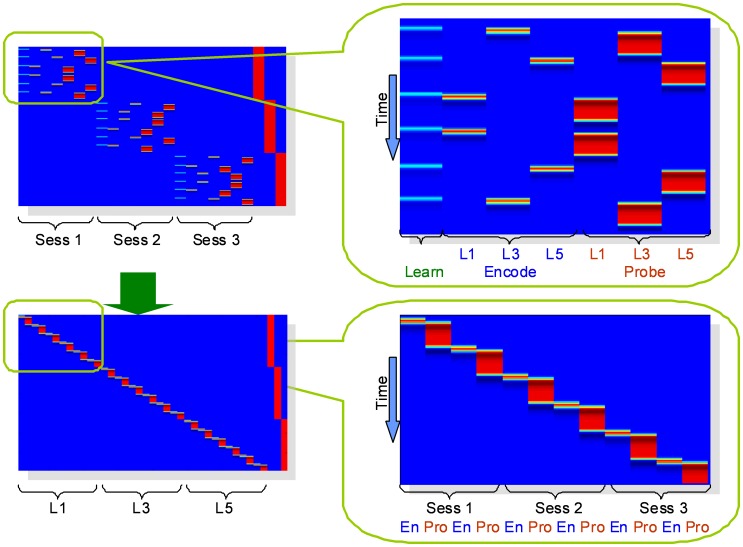
Original (top) and sorted (bottom) blocks order. Time courses sorted according to the WM loads within each run. Details are enlarged on the right.

Time courses were grouped according to WM load levels (1, 3, 5-digit), as illustrated in Figure 4. The time courses of source data were truncated into blocks based on the onset time of design matrix. Each block consisted of one encode and one probe epoch, while the learn prompt was discarded. The time courses of each six blocks with the same WM load level were then concatenated, so that the BOLD signals in each subject were separated according to different WM load levels instead of task runs. This results in three time series for each subject, corresponding to each of the 1, 3 or 5-digit condition in SIRP.

### Partial Correlation Matrices

To measure the functional connection strength between brain regions, partial correlation was adopted to construct the connectivity matrices of the networks. This approach has been used in previous small-world brain networks studies like [7], [9], [10]. Partial correlation is the correlation between two random variables, with the effect of a set of controlling random variables removed [55], [56], [57]. A partial correlation coefficient within a functional network measures the interaction between the time courses of two blocks, once these signals have been projected on the subspace orthogonal to the time courses of all other regions. Hence, it only considers the “direct correlation” between the two blocks of interest, without influence of other areas in the network [58]. By taking partial correlation instead of taking direct correlation, the inter-correlated effects within each nearby regions were removed, such that the indirect dependencies between ROI’s can be filtered out.

To denote the interaction between each ROI at a specific WM load level, three networks corresponding to each load level were built for each subject. Due to the 105 spatial blocks in the ROI, each partial correlation matrix in this study was a 105 by 105 symmetric matrix, in which each off-diagonal element *z_ij_* was the correlation coefficient between time courses in corresponding *i* ^th^ and *j* ^th^ block after filtering out the contribution of activations from all other 103 brain regions in ROI.

As the partial correlation matrices were not normally distributed, so a Fisher r to z transformation was used on each element of the matrices [7], [59].

### Constructing Brain Network

In many recent studies on brain networks, edge weights are often binarized. Binary networks are generally simpler to characterize since the null model used in statistical comparisons is more easily defined. This is achieved by specifying a weight threshold and discarding weak and non-significant edges [60]. In order to find unweighted undirected networks, we binarized the elements with a threshold *T*,
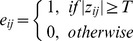
(1)where *e_ij_* is the new weight value and *z_ij_* is the old weight value in the unthresholded network.

The selection of threshold *T* will be discussed in following sections. The diagonal elements of the adjacency matrices are set to be 0 as there is no edge from a node to itself.

### Measures of Brain Functional Networks

Topological properties reveal the characteristics of connectivity in the network. Suppose we have an undirected, binarized network *G* with *N* nodes. We start with the concept of subgraph. The subgraph *G_i_* is the set of nodes that are the direct neighbors of the *i* ^th^ node. That is, every node in *G_i_* could reach the *i* ^th^ node through one edge. If there are *k* nodes, the total possible number of edges is 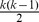.

The *degree* of a node *K_i_* represents the total number of edges connecting to a node. The degree of overall network *K_net_* is the average degree of all nodes.
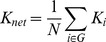
(2)


Some others prefer to use the cost (connection density) of the network instead, which is the total number of edges in a graph, divided by the maximum possible number of edges 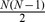:
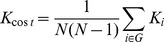
(3)


The *absolute clustering coefficient* provides a measure of functional segregation, showing the prevalence of clustered connectivity around individual nodes [1]. Locally, the clustering coefficient *C_i_* is known as the fraction of existing connections *E_i_* to the number of all possible edges in subgraph *G_i_* around one node.
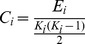
(4)


Clustering coefficient of a network *C_net_* is then derived by averaging the clustering coefficients of all nodes within the network.
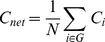
(5)


The *shortest path length* of a node pair min{*L_i, j_*} is the smallest number of edges connecting the *i* ^th^ and the *j* ^th^ node. The mean shortest path length of a node *L_i_* is the mean value of shortest path length from *i* ^th^ node to all other nodes in the network.
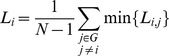
(6)


Similarly, mean shortest path length of the network *L_net_*, or *characteristic path length* [1], is the mean of shortest path length between all node pairs in the network.
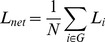
(7)


Characteristic path length reflects the average connectivity or overall routing efficiency of the network. When the network is disconnected (i.e., there are nodes in the network with no existing path to certain other nodes), the shortest path length is set to be infinity.

*Global efficiency E_global,net_* measures how efficient a network is to transfer information parallelly at a relatively low cost. It is defined as the inverse of harmonic mean of the shortest path length between each pair of nodes [61], [62], [63].
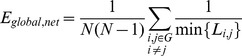
(8)


Similarly, *local efficiency E_local,net_* can be defined the same way for the subgraph *G_i_*:
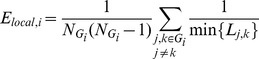
(9)

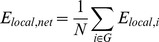
(10)


From the definition of subgraph, *G_i_* itself does contain the *i* ^th^ node. Thus, the local efficiency could be interpreted as how well the nodes in subgraph *G_i_* exchange information when the *i* ^th^ node is removed, revealing the tolerance of the network.

### Small-world Properties

Mathematically, small-world networks have similar characteristic path length but higher absolute clustering coefficients comparing to random networks [1], that is,
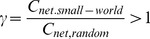
(11)

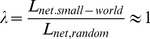
(12)The small-worldness is defined as
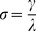
(13)which is larger than 1 for small-world network [8], [64], [65].

The measures on clustering coefficients and characteristic path length of random networks with similar degree distribution should be obtained for comparison when computing the small-worldness. Previous studies have shown that the theoretical values of these two measures are:
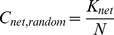
(14)

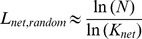
(15)where *K_net_* and *N* are the degree and total number of nodes in the existing network [8]. However, some studies have suggested that building random networks with equal (or at least equal) degree sequences as the real small-world networks may not provide valid statistical comparisons [6]. This is because theoretical random networks have Gaussian degree distributions which differ from the distributions of real networks being compared against. Therefore, to obtain a more valid comparison for each network to be measured, we built 25 random networks using Markov-chain algorithm starting from its degree distribution [2], [66], [67]. The small-worldness value for each network is then derived by averaging the 25 σ values. This method has been used in previous studies [7], [10], [68].

### Thresholding the Networks in Small-World Regime

To make the networks comparable, the thresholding condition for all networks must be uniform. The threshold values should be within a certain range to keep the network under the optimized small-world structure, same as previous small-world network studies [7], [10], [68]. First, the maximum threshold should ensure that every network is fully connected, that is, all nodes in a network could be accessible via one or multiple steps from any other nodes in the same network, or no infinite shortest path length for all nodes. At the same time, the minimum threshold must make sure that all networks hold small-world properties. Specifically, every thresholded network must have a small-worldness value of larger than 1 [63].

It is obvious that different threshold values will have a major impact on the topological properties of the thresholded networks. Because between-subject variations, and the variations in weights for each edge within the networks are both large, binarizing all networks with a uniform threshold may not be a good choice. However, leaving a same degree value for each network may keep similar structures along all subjects, and will be easier to perform comparisons between SZ and HC. A degree range was found between 19.9 and 35.0 (equivalent to cost from 0.191 to 0.337), which satisfies fully connected small-world network condition for all subjects in each of the three memory loads. Within this range, sixteen degrees values, from 19.9 to 34.9 (equivalent to cost from 0.191 to 0.336), with an increment of 1.0, were taken for multiple observations.

### Graphical and Statistical Analysis

At each of 16 degree values, an observation of network measures including clustering coefficient (*C_net_*), characteristic path length (*L_net_*), local efficiency (*E_local,net_*) and global efficiency (*E_global,net_*), was calculated for every binarized network. Site effects on individual network measures were corrected for proceeding analysis by conducting one-way analysis of variance (ANOVA). The averages of network measures were estimated over 16 observations per subject to show the overall changes across WM loads. To assess for differential effects of WM load on functional connectivity in SZ versus HC, we conducted two-way ANOVA to test the main effects of WM load x group interaction. In addition, we used two-sample t-tests to further determine group differences in functional network properties at each WM load level. Similar two sample t-test were also utilized to compare the measures in HC at WM load level 5 and those in SZ at level 3, since previous studies [69], [70], [71] have found that WM performance and prefrontal activation in HC at high WM load match those in SZ at medium WM load. Right-tailed (or left-tailed) one sample t-tests were performed on the contrasts of measures between WM loads for each group, to test the increases (or decreases) of measures as WM load level increases. During statistical tests, the averaged network measures across 16 observations were checked first. If significance exists, network measures at each observation were further looked into. False discovery rate (FDR) correction [72] was used on p-values gained from t-tests made on 16 individual observations, to control for multiple comparisons.

To evaluate how the network properties affect the actual WM performance, we used Pearson’s correlation coefficients to investigate the relationships between small-world network measures (i.e., clustering coefficients, characteristic path length, local efficiency and global efficiency) and WM behavior data (i.e. averaged reaction time of each load, which denotes the duration between subject seeing the number and pushing the button at the probe epoch). The Pearson’s correlation coefficients were also adopted to check if there are any effects on network measures from subjects’ demographic information (age, education, and handedness), the SZ’s clinical characteristics (the Scale for the Assessment of Negative Symptoms (SANS) [73] and the Scale for the Assessment of Positive Symptoms (SAPS) [74]), and head motion during the scanning.

## Results

### Behavioral Results

The group averages of the subjects’ accuracy and reaction time by level are shown in Table 2, and the trends are plotted in Figure S1. Reaction times for each subject were averaged on correct trials only. Subjects showed a reasonably high percentage of correct responses (mean accuracy ≥95% for all WM loads). Both groups showed decreased accuracy and increased reaction time as WM load increased. Two-way ANOVA test indicated group and load effect (*F* = 20.11, *p* = 1.2×10^−5^ for group effect, and for *F* = 41.91, *p*<0.0001 load effect) on RT, and group effect (*F* = 17.72, *p*<0.0001) on accuracy. No interaction between group and load was found in both RT and accuracy.

**Table 2:** Subjects’ Performance on SIRP.

	HC	SZ	Two sample(t-value/p-value)
**Load Level 1**			
** Accuracy (%)**	99.2±1.1	96.7±5.9	2.42/0.02
** Reaction time (msec)**	550±74	594±85	2.31/0.02
**Load Level 3**			
** Accuracy (%)**	98.4±1.6	95.5±6.1	2.79/0.01
** Reaction time (msec)**	636±84	702±116	2.91/<0.01
**Load Level 5**			
** Accuracy (%)**	97.3±2.6	95.4±4.9	2.03/0.04
** Reaction time (msec)**	691±98	764±137	2.58/0.01
**All load levels Mean**			
** Accuracy (%)**	98.3±1.5	96.0±5.0	2.77/0.01
** Reaction time (msec)**	626±81	685±110	2.72/0.01

### Network Measures at Each WM Load

Two-way ANOVAs on averaged network measures showed significant group by load interaction in averaged *C_net_* and *E_local,net_* (*p*<0.05). Among individual observations, marginally significant group by load interactions (*p*<0.1, FDR corrected) were found for *C_net_* and *E_local,net_* at most of the observations.

For averaged network measures across 16 degree points at single WM load, the t-test indicated group differences (*p*<0.05) at WM load level 3 on all four measures, clustering coefficient *C_net_*, characteristic path length *L_net_*, local efficiency *E_local,net_* and global efficiency *E_global,net_*. No significant group differences were found at WM load levels 1 or 5.

**Figure 5:**
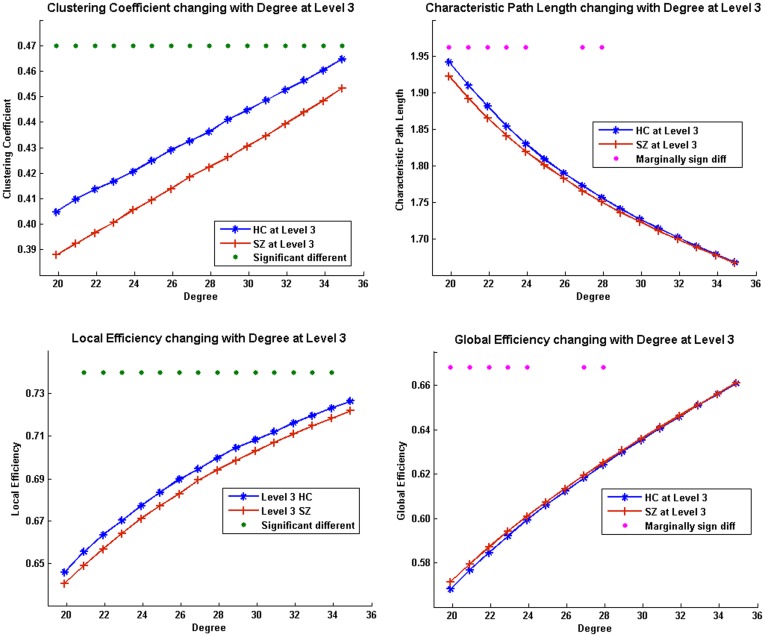
Group comparison on network measures at medium load. Network measures on individual observations as a function of degree between groups on WM load level 3. Green dots above indicate significant group difference (*p*<0.05, FDR corrected) and pink dots above indicate marginally group difference (*p*<0.1, FDR corrected) between HC and SZ at that observation.

Next, we examined individual observations at each degree value *K_net_* in the small-world regime within WM load level 3 (Figure 5). Throughout 16 observations, two sample t-tests showed all *C_net_* and most of *E_local,net_* had significant group differences (*p*<0.05, FDR corrected). Some *L_net_* and *E_global,net_* had group differences that approached but did not achieve statistical significance (*p*<0.1, FDR corrected). When degree *K_net_* increases (i.e., more edges being added into the network), *C_net_*, *E_local,net_*, and *E_global,net_* also increase whereas *L_net_* decreases. In all observations at WM load level 3, networks in SZ had lower *C_net_*, *L_net_*, *E_local,net_* and higher *E_global,net_* than HC.

**Figure 6:**
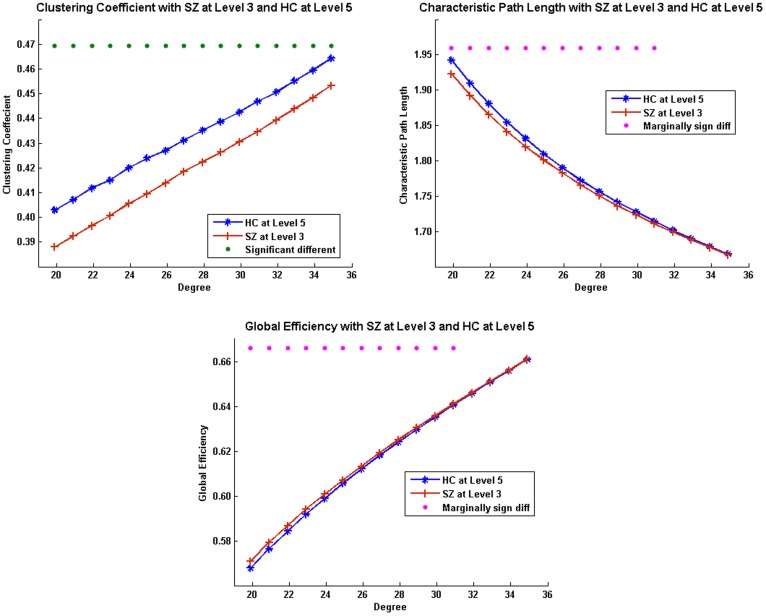
Network measures between SZ at high load and HC at medium load. Green dots above indicate significant group difference (*p*<0.05, FDR corrected) and pink dots above indicate marginally group difference (*p*<0.1, FDR corrected) between HC and SZ at that observation.

In contrasting WM loads between SZ at level 3 and HC at level 5, we still found significant group differences (*p*<0.05) in averaged *C_net_*, *L_net_* and *E_global,net_*. Among individual observations, significant group differences (*p*<0.05, FDR corrected) existed in *C_net_* at all observations, and marginal significant group differences (*p*<0.1, FDR corrected) in *L_net_* and *E_global,net_* at most of the observations (Figure 6).

### Network Measures Change at Different WM Loads

**Figure 7:**
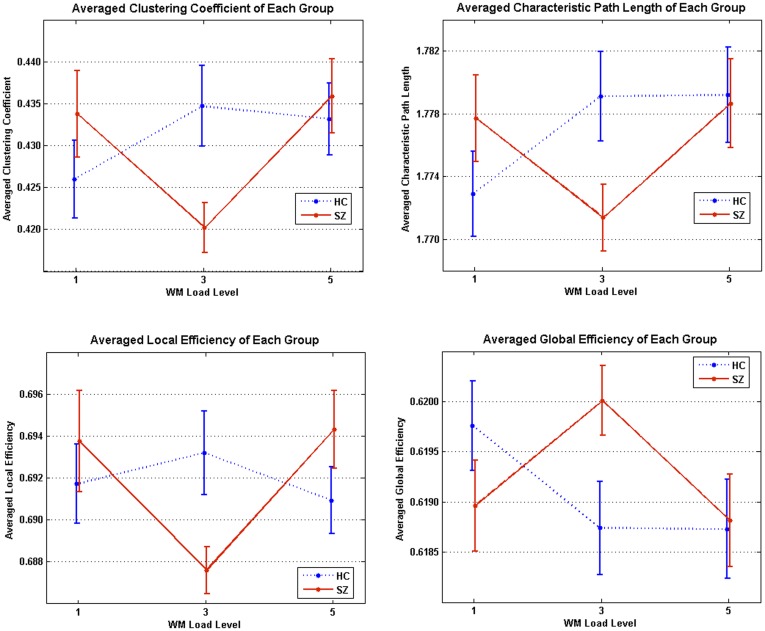
Averaged network measures changes across three load levels in HC and SZ. Solid lines between WM load levels indicate significant increases/decreases (*p*<0.05), and dotted lines indicate no significant changes when WM load levels increases.

When WM load increases, changes in network measures showed different patterns in each group. Figure 7 showed a general trend of averaged network measures change across different WM loads.

In HC, there were no significant changes in network measures between different WM loads in HC. Network measures in SZ, however, revealed significant changes across three WM loads. All four averaged measures in patients had no significant different between WM load level 1 and 5, but altered significantly (*p*<0.05) in level 3. In individual observations, *C_net_* and *E_local,net_* in all observations in SZ showed significant decreases (*p*<0.05, FDR corrected) from WM load level 1 to level 3, and significant increases (*p*<0.05, FDR corrected) from WM load level 3 to level 5. At the same time, *L_net_* and *E_global,net_* in SZ showed marginal significant changes (*p*<0.1, FDR corrected) from WM load level 1 to level 3 in some (5 out of 16) observations, and marginal significant changes (*p*<0.1, FDR corrected) from WM load level 3 to level 5 in most (14 out of 16) observations.

### Correlation between Network Measures and Behavioral Data

**Figure 8:**
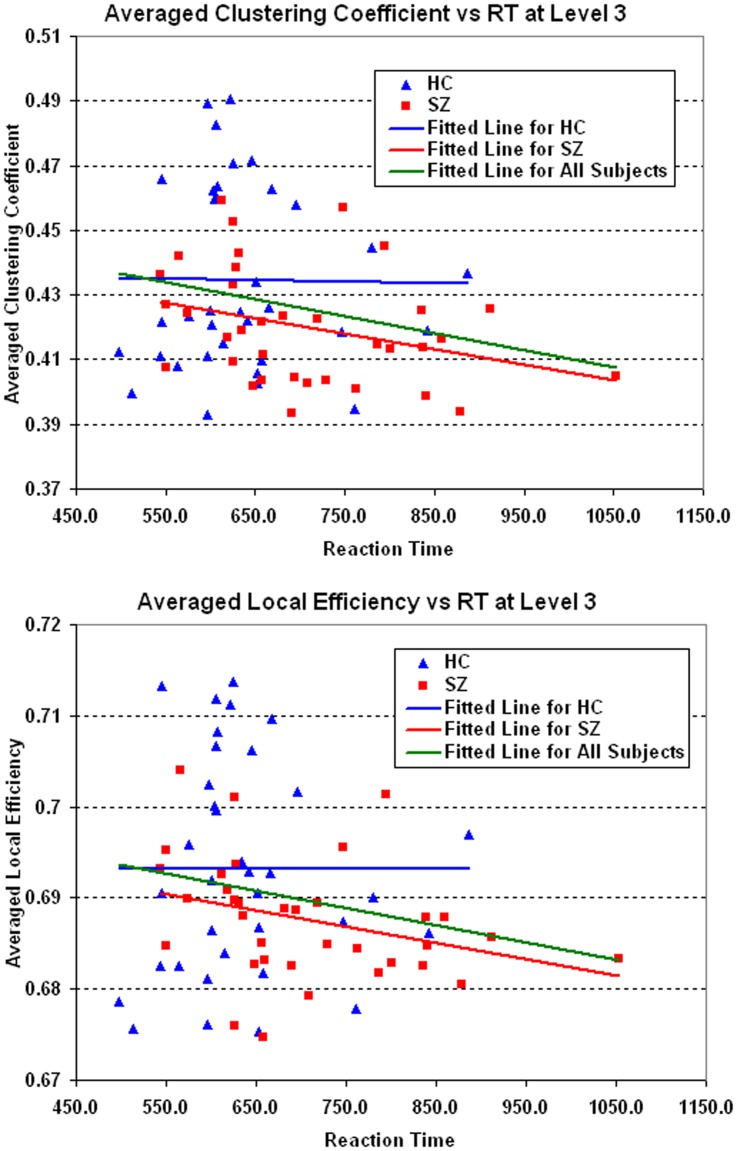
Scatter Plots of averaged clustering coefficients and local efficiency against RT at medium load. Scatter plots with trend lines showing averaged *C_net_* and *E_local,net_* as function of reaction time in all subjects and each group. Significant negative correlation (*p*<0.05) was found between reaction time and averaged *C_net_* and *E_local,net_* for all subjects (green line).

Significant negative correlations (*p*<0.05) were found between reaction time and averaged network measures *C_net_* and *E_local,net_* for all subjects at WM load level 3. Patterns are shown in Figure 8. No correlations were found in other WM loads.

In individual observations, lower *C_net_* and *E_local,net_* were also predicative of longer reaction times at medium WM load level. All 16 observations of *C_net_* and most (11 out of 16) observations *E_local,net_* showed significant correlations (*p*<0.05, FDR corrected).

Within each group, there were no associations of the measures with reaction time in HC. In SZ, the correlations at more than half (9 out of 16) of observations approached but did not achieve statistical significance (*p*<0.1, FDR corrected).

### Effects from Demographics, Clinical Characteristics and Head Motion

No statistically significant effects (*p*<0.05) were found on network measures from either demographics in both groups or clinical characteristics data in SZ.

On each of the six parameters of head motion (translation and rotation on each axis) outputs of the SPM realignment parameters, no statistically significant group differences (*p*<0.05) showed between HC and SZ. Four measurements of head motion (mean motion, maximum motion, mean rotation, and number of movements) during the entire scanning process were further calculated using the translation and rotation parameters from the rigid body correction [48], [75]. Significant group differences (*p*<0.05) were found in mean motion, mean rotation, and number of movements. However, there is no statistically significant correlation between any head motion measurements and network measures.

## Discussion

In this fMRI study, topological and efficiency properties of WM-related networks were examined for both HC and SZ groups. First, group ICA was performed to detect task-related networks. Then partial correlation was used to generate adjacency matrices on 105 WM-related regions for each subject per WM load level. The networks were thresholded within the small-world regime. Statistical tests on network measures taken at 16 different degrees showed significant altered topology and efficiency in SZ at medium WM load. The pattern of altered network measures in SZ at medium WM load is similar to findings in [26], [76], which showed altered DLPFC activation in SZ at medium WM load during a similar SIRP performance. The subjects’ demographics, clinical characteristics and head motion during scanning had no significant effects on network measures.

The findings indicate that network measures differed significantly in SZ at the medium WM load level during the SIRP performance. For both topological and efficiency measures, we found that most group differences were between SZ at WM load level 3 and healthy control at either level 3 or level 5. Clustering coefficients, characteristic path lengths and local efficiency were lower for people with SZ while global efficiency was higher for that group.

Topologically, clustering coefficients equivalent to the fraction of the node’s neighbors that are also connected with each other [1], which reveals the abilities for specialized processing to occur within densely interconnected groups of regions in brain [60]. Lower clustering coefficients in SZ for intermediate working memory loads indicate that the networks had fewer local functional interconnections, and thus were less efficient for local information transfer. Functional dysconnectivity found in SZ here is also consistent with the facts found in [69], which confirmed that SZ activated fewer DLPFC voxels in common than HC, even when task performance was matched with HC.

Local efficiency reflects the fault tolerance of the network system, or the efficiency of communication between the first neighbors of a node when it is removed [61]. Brain networks with high clustering and high local efficiency are robust in local information processing even if some neurons are inefficient or damaged [77]. The low local efficiency and low clustering in SZ at WM load level 3 we found in the current study suggests that the network of task-related brain regions in SZ had lower fault tolerance (i.e., more vulnerable) locally than HC. Our findings on reduced local efficiency and clustering are also consistent with prior fMRI and EEG studies on functional brain networks in SZ [7], [11], [31], [78], [79], [80].

In general, subjects with longer reaction time also had lower clustering coefficient and local efficiency at WM load level 3, as seen in the negative correlation between reaction time and those two measures. The less clustered structure and lower efficiency of task-related networks in SZ may affect the performance to accomplish the task.

In brain networks, the identified paths show potential routes of information flow between pairs of brain regions [60]. Characteristic path length is a measurement on the extent of average connectivity or overall routing efficiency of the network. Global efficiency in graph system is the efficiency of a parallel system, where all the nodes in the network exchange information concurrently [61]. Networks with shorter characteristic path length and high global efficiency are of significance in minimizing noise, shortening signaling delay and increasing synchrony [81]. Shorter path lengths between nodes have also been shown to promote effective interactions across different cortical regions [3], [63]. Among SZ, the networks related to intermediate WM loads had shorter path lengths and higher global efficiency than those in HC. Because of abnormalities within brain regions like DLPFC, SZ may need to compensate for this impairment by involving more brain regions concurrently so as to achieve comparable WM performance, resulting shorter path lengths and higher global efficiency. The phenomena that SZ shows reduced clustering but globally efficient and robust were also found in previous network studies [80], [82]. Lynall et al. in [80] suggested that reduced local dominance will generally be offset by greater network robustness in SZ. In contrast, activations in HC subjects at WM medium load levels concentrated more on certain brain areas, even at the cost of low global efficiency within the network.

In a previous WM study on SZ which utilized the same version of the SIRP [26], Potkin et al. also found that the medium WM load was most responsible for significant group differences in the DLPFC activation. They attributed these differences to the “inefficiency” of this brain region that might not be directly caused by increases in WM load. A multivariate analysis using Partial Least Squares on the same data [76], Kim et al. showed that other areas in frontal lobe, pre and post central gyrus, and the angular gyrus showed a similar pattern for the probe condition, while the visual cortex showed a pattern of greater activation in the SZ subjects in the encoding of the medium load WM condition rather than during the probe epoch. [37] used ICA on a SIRP dataset of which this was a subset, and identified a frontal/parietal network which showed more activation in SZ subjects in the moderate load conditions as well. Our small-world network analysis did not distinguish encode from probe responses in determining the network edges, but considered the correlations among the 105 brain regions at each WM load. Although encoding and maintaining information involve different psychological processes, they are both affected by load and are needed to perform a WM task. Many previous studies on WM combine them, for example, N-back paradigm [35], looks into the two psychological processes in subjects simultaneously. Brain connectivity associated with both encode and probe (a.k.a. retrieval) conditions have also been combined in previous SIRP studies [19], [83]. Our combination of encode and probe still identified the moderate WM load level as the condition in which the network measures in SZ were different from those in HC.

Depending on the level of demands on working memory, different physiological responses showed up in each group to accomplish the task. In HC, we found no significant changes across different WM load. Although previous studies in WM like [15], [69], [70] have shown increased BOLD activities as WM load increases within subjects’ capacities, the topology and efficiency of functional networks in HC remain stable.

In contrast, the small-world network measures in SZ showed a different pattern of responses with increasing WM loads, consistent with the inverted-U function relating fMRI signal to WM load [16], [84]. There were significant differences in small-world network measures across different WM load levels among patients. The fact that local clustering coefficient and local efficiency in SZ during WM load level 1 were close to those in HC at WM load level 3, indicated that more effort may have been used by patients to perform the low difficulty task [18]. As WM load level increased from low to medium, the clustering coefficients and local efficiency dropped significantly, but path length reduced and global efficiency rose. This implies that connectivity tends to be more spread across the brain regions included in analysis, or that more voxels in brain regions for SZ were utilized concurrently to perform the task, as discussed above. When tasked with the medium WM load, functional brain networks adapt to the increased WM demands by increasing global integration and efficiency [33], [85], [86], [87]. However, when the difficulty level increased further from medium to high, the clustering coefficients and local efficiency returned to values comparable to those in the low difficulty condition. These changes suggest that the highest memory load may have approached SZ’s WM capacity, and the patients will be no longer able to adequately perform more difficult tasks [16], [26], [37].

In this paper, we focused on the small world network of task-related regions. In order to avoid certain bias, we also tried another approach by using components of the whole brain that includes non-task-related regions, in which group ICA is performed on the same data, generating 80 components. After eliminating artifactual components (by visual inspection) which contain obvious skull edge effects or ventricles, 39 components were chosen, each as one node, to build the connectivity network using the same method mentioned above. The trends we observed were consistent with the main results discussed above, but with weaker between-group differences. Since the selected components included both task-related and non-task-related ICs, our results suggested that the non-task-related brain regions contributed less to the WM task, but may add more individual variation to the network.

There are several methodological issues that should be considered in this study. First, we used group ICA to find the task-related region, and divided the region into 105 contiguous voxel clusters, corresponding to nodes in the brain networks. Although cluster size and the randomness generated from group ICA may impact network structures, we have averaged the results of group ICA from multiple runs so as to mitigate against this possibility. Secondly, we used partial correlation on BOLD signals between brain regions to construct brain networks, which may be affected by the operation of truncating and reordering the time courses to separate WM loads. There are other methods that may be worth trying in future studies. Pearson’s correlation and partial correlation of time series between different brain regions are commonly used in fMRI networks [4], [7], [9], [10], [11], [29]. However, a recent study [86] built task-related functional networks from the correlations of beta values derived from regression against stimuli. Also, the networks we analyzed are undirected and binarized. Future studies may perform a weighted network analysis which could supply more information as has been done in two recent studies [88], [89]. Another concern is that patients had received antipsychotic treatment, but the detailed medication history was not available for all subjects recruited in this study. This raises the potential confound that antipsychotics may contribute to the differences in graph parameters among patients.

### Conclusion

In this paper, we examined the differences between healthy controls (HC) and people with schizophrenia (SZ) on topological properties of small-world networks that were derived from fMRI data acquired during working memory performance. Brain networks were constructed for each subject based on the functional connectivity between brain regions constrained to components of task-related brain areas for each WM load level. The constructed brain networks were thresholded to derive small-world networks with a series of constant number of edges for all subjects. Next, topological and efficiency measures for brain networks in both groups were generated. Results showed that topologies and efficiencies of functional networks in HC were stable as WM load increases, while network measures in SZ altered significantly at medium WM load. The network measures implied brain connectivity in SZ was more diffuse and less strongly linked locally in functional network at intermediate level of WM when compared to HC. The differential local and global patterns of connectivity and efficiency for people with SZ across levels of WM load indicate that patients are inefficient and variable in response to WM load increase, comparing to stable highly clustered network topologies in HC. Sophisticated graph network measures provide a means of characterizing the effects of dysfunctional neural circuitry and variations in impaired connectivity across levels of dysconnectivity working memory demands in SZ [15], [18], [26], [69]. The present findings also suggest that graph theoretic descriptions of neural connectivity may help isolate the conditions under which neural contributions to working memory deficits are most evident in the disorder.

## Supporting Information

Figure S1 **The behavioral data (accuracy and reaction time) verses WM load levels for each group.**
(TIF)
