# Supplementary figures and images for: Altered Small-World Brain Networks in Schizophrenia Patients during Working Memory Performance
Source: PLoS One. 2012 Jun 6;7(6):e38195. doi: 10.1371/journal.pone.0038195 (PMC3368895; doi:10.1371/journal.pone.0038195)

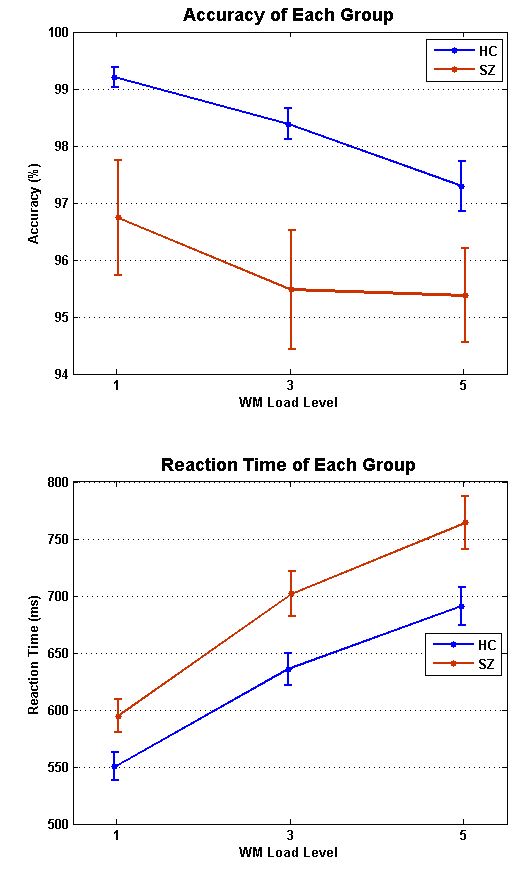

Supplement: Figure S1 — The behavioral data (accuracy and reaction time) verses WM load levels for each group. (TIF) [file pone.0038195.s001.tif]
